# Genetically Engineered Ascorbic acid-deficient Live Mutants of Leishmania donovani induce long lasting Protective Immunity against Visceral Leishmaniasis

**DOI:** 10.1038/srep10706

**Published:** 2015-06-02

**Authors:** Sneha Anand, Rentala Madhubala

**Affiliations:** 1 School of Life Sciences, Jawaharlal Nehru University, New Delhi 110067, India

## Abstract

Visceral leishmaniasis caused by L*eishmania donovani* is the most severe systemic form of the disease. There are still no vaccines available for humans and there are limitations associated with the current therapeutic regimens for leishmaniasis. Recently, we reported functional importance of Arabino-1, 4-lactone oxidase (ALO) enzyme from *L. donovani* involved in ascorbate biosynthesis pathway. In this study, we have shown that *ΔALO* parasites do not affect the ability of null mutants to invade visceral organs but severely impair parasite persistence beyond 16 week in BALB/c mice and hence are safe as an immunogen. Both short term (5 week) and long term (20 week) immunization with *ΔALO* parasites conferred sustained protection against virulent challenge in BALB/c mice, activated splenocytes and resulted in induction of pro-inflammatory cytokine response. Protection in immunized mice after challenge correlated with the stimulation of IFN-γ producing CD4^+^ and CD8^+^ T cells. Antigen-mediated cell immunity correlated with robust nitrite and superoxide generation, macrophage-derived oxidants critical in controlling *Leishmania* infection. Our data shows that live attenuated *ΔALO* parasites are safe, induce protective immunity and can provide sustained protection against *Leishmania donovani.* We further conclude that the parasites attenuated in their anti-oxidative defence mechanism can be exploited as vaccine candidates.

Visceral leishmaniasis (VL) is a major public health problem in tropical and subtropical countries. The disease is caused by an intracellular protozoan parasite of the *Leishmania donovani*/*L. infantum*/*L. chagasi* complex. Availability of a limited arsenal of anti-protozoal drugs and emergence of drug resistance has worsened the situation. Unfortunately, no effective vaccine has been found against leishmaniasis despite intensive efforts been put into vaccine development.

Host resistance to *Leishmania* infection is mediated by cellular immune responses leading to macrophage activation and parasite killing. Immunity to leishmaniasis primarily involves a Th1 response characterized by production of IL-12 and IFN-γ^1^,^2^. These two cytokines drive the effector functions of macrophages and trigger a Th1 immune response^3^. The clear Th1/Th2 dichotomy established for Cutaneous Leishmaniasis (CL) has been questioned in VL^4^. In general it is the inability to mount Th1 response rather than the presence of Th2 response which determines disease susceptibility in VL^4^. Thus one goal of vaccine development is evoking a protective Th1 response against parasite antigens^5^.

Vaccines based on either killed parasites or recombinant DNA and protein vaccines are ineffective as a consequence of the short-term immunity they induce^6^. Another approach is to develop live vaccines for visceral form of leishmaniasis. This involves utilization of non pathogenic *Leishmania* species, such as a recombinant strain of lizard parasite *L. tarentolae*, expressing immunogenic components of *L. infantum*^7^,^8^. Furthermore, live *Salmonella* and *Lactococcus* expressing exogenous antigens have also been utilized to develop effective vaccines against leishmaniasis^9^. Several studies in mice indicate that parasite persistence is important to maintain durable, anti-*Leishmania* memory responses^6^,^10^. These findings have led to the exploration of live, genetically modified-parasites as an appealing strategy for developing anti-*Leishmania* vaccines^4^,^11^.

More recently, the ability to manipulate the *Leishmania* genome to create genetically modified parasites by eliminating genes essential for virulence revives the potential of live attenuated parasite vaccine and can be a powerful tool for developing new generation vaccines against leishmaniasis^11^,^12^. Among the vaccination studies in VL, immunization with a *L. donovani* strain deleted for biopterin transporter (BT1) was protective for mice^13^. Replication deficient *L. donovani* null mutant generated by deletion of centrin gene was also found to be protective against homologous and heterologous challenges in mice^14^. More recently *L. donovani* mutant deficient for amastigote specific p27 gene was found to be safe and conferred cross protection against *L. major* and *L. braziliensis*^15^. Partial knockout parasites for the A2-A2rel gene cluster in *L. donovani*^16^ and SIR2 gene in *L. infantum*^17^ have also been shown to induce protection against virulent challenge in BALB/c mice. However, safety of such mutants cannot be warranted because they still carry a wild type allele and could cause disease^16^,^17^. Hence it is critical to develop attenuated lines through complete gene knockouts that generate avirulent organisms with reduced risk of reactivation.

Recently, we reported generation of a homozygous null mutant *L. donovani* cell line (*∆ALO)* devoid of Arabino-1, 4-lactone oxidase enzyme that catalyzes the last step in ascorbate biosynthesis pathway^18^. Loss of the *ALO* gene from *Leishmania* abrogated the production of ascorbate, an important antioxidant, consequently resulting in impaired infectivity *in-vivo* in susceptible BALB/c mice. In this study we clearly demonstrate that *∆ALO* parasites are able to invade but not persist in visceral organs and vaccination with *ΔALO* attenuated strain protects BALB/c mice against virulent *L. donovani* challenge. Furthermore a strong correlation was found between elimination of parasites and an increased Th1 immune response. Our data shows that genetically modified live attenuated *ΔALO* parasites, can elicit an effective cell-mediated protective immune response against *L. donovani.*

## Results

### Live attenuated ΔALO parasites show reduced infectivity and limited persistence in mice

**Figure 1:**
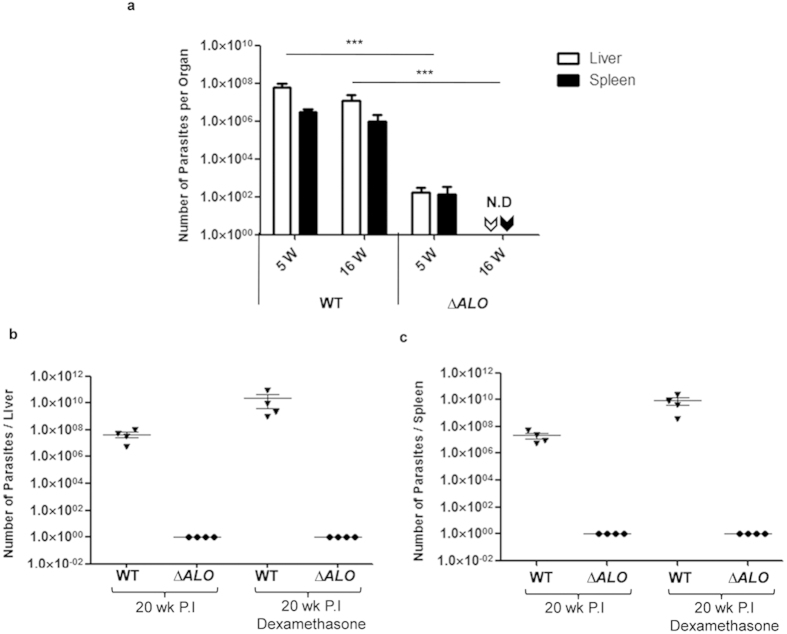
Avirulent properties of *ALO* null mutant of *L. donovani* parasites (*ΔALO*) in BALB/c mice. (**a**) Survival of WT or *ΔALO* parasites in liver and spleen of BALB/c mice. Parasite load in organs of mice infected with promastigotes of either WT or *ΔALO* parasites were measured at 5 or 16 wk post-infection by serial dilution method. Data presented are the average number of parasites per organ in liver or spleen. Error bars indicate the standard error. Effect of immunosuppressive drug dexamethasone on infected mice. Parasite load was measured in liver (**b**) and spleen (**c**) of mice infected with WT or *ΔALO* parasites for 20 weeks post-infection and treated with dexamethasone. The data presented are representative of three independent experiments with similar results and four mice in each group. Mean and SEM of each group are shown. *p < 0.05; **p < 0.01; ***p < 0.001 by one way ANOVA with Bonferroni’s multiple comparison post-test. ND, Not detected.

We have previously reported that *ΔALO* mutant parasites are attenuated for their infectivity in susceptible BALB/c mice^18^. This incapacity was shown to be a direct consequence of ALO deficiency^18^. To further analyze long term persistence of *ΔALO* parasites in liver and spleen of mice, groups of six BALB/c mice were injected i.v. with stationary phase *∆ALO* or WT parasites and the parasite burden was monitored at 5 wk and 16 wk post-infection by limiting dilution method^19^. The parasite burden remained significantly high in both liver and spleen of WT-infected mice for up to the 16^th^ week reflecting normal course of infection (Fig. 1a). On the other hand, significantly less *∆ALO* parasites were detected 5 wk post-infection in spleen (*p* < 0.001), and liver (*p* < 0.001), and by 16 wk, no live parasites were detected in both organs (Fig. 1a). We further confirmed these results by real-time PCR analysis of parasite minicircle DNA; using DNA from spleen and liver of mice infected either with WT or *ΔALO* parasites (Supplementary Table S1). After 16 wk mice infected with *ΔALO* showed complete clearance of parasites from both spleen and liver thereby suggesting absence of *ΔALO* parasites in mice at this time point. Our results clearly indicate that *∆ALO* deletion mutants do not affect the ability of null mutants to invade visceral organs but severely impair parasite persistence in these organs.

Parasite survival was further assessed by immunosuppressing the animals with dexamethasone, a known immune suppressor. These studies were essential to show that *∆ALO* mutant parasites are safe and do not persist in visceral organs beyond 20 wk post infection. In order to allow proliferation of any residual parasites, WT or *∆ALO* parasite–infected mice were treated with dexamethasone. Dexamethasone treatment in the WT-infected group showed enhancement of parasite growth in both liver (Fig. 1b) and spleen (Fig. 1c) by ~2 log fold at 20 wk post-infection. However, in *∆ALO*-infected mice at 20 wk post-infection, with or without dexamethasone treatment, we did not observe any parasites in the liver (Fig. 1b) and spleen (Fig. 1c). These observations confirm that *∆ALO* is a safe immunogen and does not persist 20 wk post-infection.

### Immunization with ΔALO parasites provides protection against challenge with virulent Leishmania

**Figure 2:**
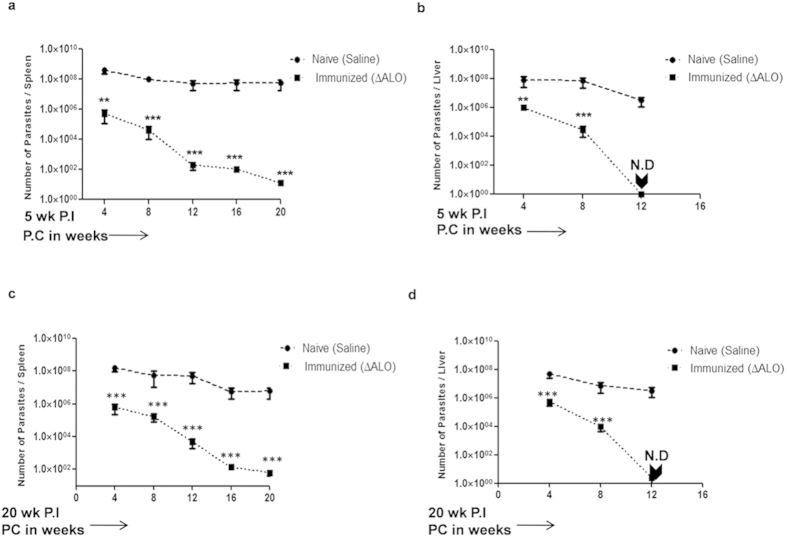
Immunoprotective properties of *ΔALO* parasites in BALB/c mice. Protection of *ΔALO* vaccinated BALB/c mice against virulent challenge was evaluated. Mice vaccinated with *ΔALO* parasites were challenged 5 wk P.I with virulent WT *L. donovani* and the parasite burdens from the spleen (**a**) and liver (**b**) were measured after different time periods P.C. Long-term effect of immunization was evaluated in 20 wk P.I mice challenged with virulent WT *L. donovani*. Parasite burden was monitored in spleen (**c**) and liver (**d**) after different time periods P.C. Mean and SEM of five mice in each group are shown. Data presented are the average number of parasites per organ in liver or spleen. Error bars indicate the standard error. The experiment was repeated three times with similar results. *p < 0.05; **p < 0.01; ***p < 0.001 by one way ANOVA with Bonferroni’s multiple comparison post-test. ND. Not Detected, P.C. Postchallenge.

Since *ΔALO* parasites are present for at least 5 wk in spleen and liver (Fig. 1a) before being cleared therefore the ability of these parasites to protect against *Leishmania* challenge was determined in mice immunized for 5 wk. To determine the protective efficacy of *ΔALO* immunization, age-matched naive BALB/c mice were injected with saline or *ΔALO* parasites i.v. At 5 wk postimmunization (P.I), mice were challenged with 1 × 10^7^ virulent *L. donovani* promastigotes. Assessment and comparison of parasite burden in visceral organs of mice was done 4, 8 12, 16 and 20 wk postchallenge (P.C) in non-immunized naive challenged mice and *ΔALO* immunized challenged mice by limiting dilution method (Fig. 2). Continuous reduction of parasite burden in both spleen (Fig. 2a) and liver (Fig. 2b) of *ΔALO* immunized and challenged mice were seen at all time points P.C. Importantly, *ΔALO* immunized and challenged mice were able to control the infection in 12 wk by completely eliminating the parasites from liver (Fig. 2b) and reducing parasite load in spleen (Fig. 2a) by more than 90% (~5.5-log fold) as compared to the naive challenged mice. The parasite burden in visceral organs of non-immunized naive challenged mice remained high at all time points. Since complete clearance of parasite was not observed in spleen at 12 wk P.C, the parasite load was further monitored up to 20 wk P.C. Even at 20 wk P.C sterile protection was not obtained in spleen but the parasite load reduced further (~1 log fold) when compared to 16 wk *ΔALO* immunized and challenged mice (Fig. 2a,c).

One of the main criteria of a live attenuated vaccine is to provide protection even after clearance of attenuated parasites from visceral organs. Thus, in order to evaluate the ability of *ΔALO* parasites to confer long-term protection, mice were challenged with virulent *L. donovani* parasites 20 wk following *ΔALO* immunization, and the parasite load was evaluated at various time periods P.C (Fig. 2c,d). At 12 wk P.C, 20 wk immunized mice showed significant protection, with undetectable parasite in liver and ~5-log fold reduced parasite burden in spleen. A further reduction in parasite load was observed in the spleen at 20 wk P.C. Thus, the parasite burden in the spleen and liver indicated that the level of protection in both immunized groups (5 wk and 20 wk) was similar. Overall these results suggest that immunization with *ΔALO* parasites confers strong and sustained protection against virulent challenge even at 20 wk P.I. A confirmatory real time PCR analysis also confirmed the presence or absence of parasite DNA in spleen and liver of *ΔALO* immunized challenged mice (Supplementary Table S2).

### Immunization with ΔALO induces a protective Th1 immune response upon challenge with virulent L. donovani

The characteristic immunological feature of active VL is the absence of parasite-specific cell-mediated immune responses^20^. Restoration of antigen induced immune response is imperative for effective vaccine-induced immunity. Also, in *Leishmania* infection production of Th1-related cytokines correlates with host control of parasite burden and clinical cure^21^. Th1 immunity is marked by secretion of cytokines such as IFN-γ, IL-12, TNF-α which are activators of cell-mediated immunity^22^,^23^ while Th2 cytokines like IL-4 and -10, promote humoral responses^24^,^25^.

**Figure 3:**
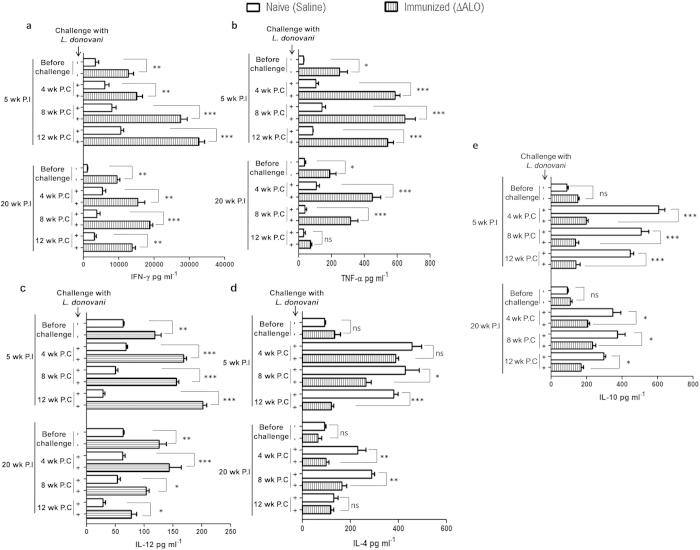
Cytokine production from *ΔALO* immunized and naive mice in response to *Leishmania* SLA, pre and postchallenge. Cytokine levels of IFN-γ (**a**) TNF-α (**b**) IL-12 (**c**) IL-4 (**d**), and IL-10 (**e**) were determined in splenocyte cultures from 5 wk or 20 wk *ΔALO* immunized and saline injected mice (naive) before and after various periods of challenge. Mean and SEM of five mice in each group are shown. The experiment was repeated three times with similar results. ns indicates p-value non significant; *p < 0.05; **p < 0.01; ***p < 0.001 by one way ANOVA with Bonferroni’s multiple comparison *p*ost-test. P.C. postchallenge. P.I. postimmunization.

To understand the nature of immune response generated by *ΔALO* immunization, we compared the levels of various cytokines secreted by splenocytes isolated from naive mice, *ΔALO* immunized, non-immunized naive challenged mice and *ΔALO* immunized challenged mice (Fig. 3). Cytokine response was characterized for both 5 wk and 20 wk (Fig. 3a–e) P.I mice before and after various periods of challenge. Elevated levels of IFN-γ (~3 fold), TNF-α (~6 fold) and IL-12 (~2 fold) was observed in the splenocytes of 5 wk as well as 20 wk (Fig. 3a,b,c) *ΔALO* immunized mice in comparison to naive control groups before challenge, Th2 cytokines like IL-4 (Fig. 3d) and IL-10 (Fig. 3e) remained similar to naive controls in both immunization (5 wk and 20 wk) groups. Thus, suggesting that *ΔALO* immunization skews immune responses towards a Th1 phenotype.

The status of these cytokines was further assessed in both 5 wk and 20 wk P.I mice, challenged for different time periods. Five wk *ΔALO* immunized and challenged mice showed ~6 fold and ~3 fold higher levels of TNF-α (Fig. 3b) and IL-12 (Fig. 3c) respectively at all time periods as compared to non-immunized naive challenged mice. IFN-γ levels showed a progressive increase in the 5 wk *ΔALO* immunized and challenged mice over time; with maximum levels being detected at 12 wk P.C (Fig. 3a). IFN-γ production increased ~2 fold at 4 wk P.C and further by ~3 fold at 12 wk P.C as compared to non-immunized naive challenged mice (Fig. 3a).

Similar analysis with spleens of 20 wk P.I mice following various periods of challenge also showed an overall Th1 dominated immune response (Fig. 3a,b,c). Of interest, in the 20 wk P.I and challenged group TNF-α (Fig. 3b) and IL-12 (Fig. 3c) expression showed a progressive decrease with time. Higher levels of IFN-γ were observed at 4 (~3 fold) and 8 wk (~4 fold) P.C as compared to non-immunized naive challenged controls (Fig. 3a). As opposed to initial periods after challenge, IFN-γ production decreased in 12 wk P.C group. A ~2.5 fold higher secretion of IFN-γ was detected in the culture supernatants of 20 wk immunized and challenged mice at 12 wk P.C as compared to non-immunized naive challenged controls (Fig. 3a).

In contrast, *ΔALO* immunization was found to inhibit the production of disease-promoting Th1-suppressive cytokine IL-10 and the Th2 signature cytokine IL-4 (Fig. 3d,e). Though the role of IL-4 in VL still awaits clarification^26^ but IL-10 has been reported to play suppressive role in *Leishmania* infections^27^,^28^,^29^. A decrease in IL-10 levels was observed in both the immunization groups (~3 fold for 5 wk immunized group and ~2 fold for 20 wk immunized group) at all time points (4 wk, 8 wk and 12 wk) P.C as compared to non-immunized naive challenged mice (Fig. 3e). Initially in 5 wk immunized group, no difference in IL-4 levels was observed between *ΔALO* immunized and challenged mice and non-immunized naive challenged mice, but subsequently 12 wk post-infection ~3 fold less IL-4 was detected in *ΔALO* immunized and challenged mice as compared to non-immunized naive challenged mice (Fig. 3d). A ~2 fold decrease in IL-4 expression was observed in 20 wk P.I and challenged mice as compared with non-immunized naive challenged mice at 4 and 8 wk P.C (Fig. 3d).

Thus, it can be assumed that the protection observed after vaccination with live attenuated *ΔALO* strain is associated with increased Th1 immune response and a concomitant down regulation of Th2 immune response.

### Immunization with ΔALO stimulates Leishmania-specific splenocyte proliferation and T-cell immune response

VL is associated with a marked depression of T cell responses, characterized by impairment of T-cell proliferation^30^ and absence of IFN-γ production by lymphocytes on *in-vitro* stimulation with *Leishmania* SLA^31^. Therefore for active protection against the disease an antileishmanial vaccine should be able to restore T-cell responses.

**Figure 4:**
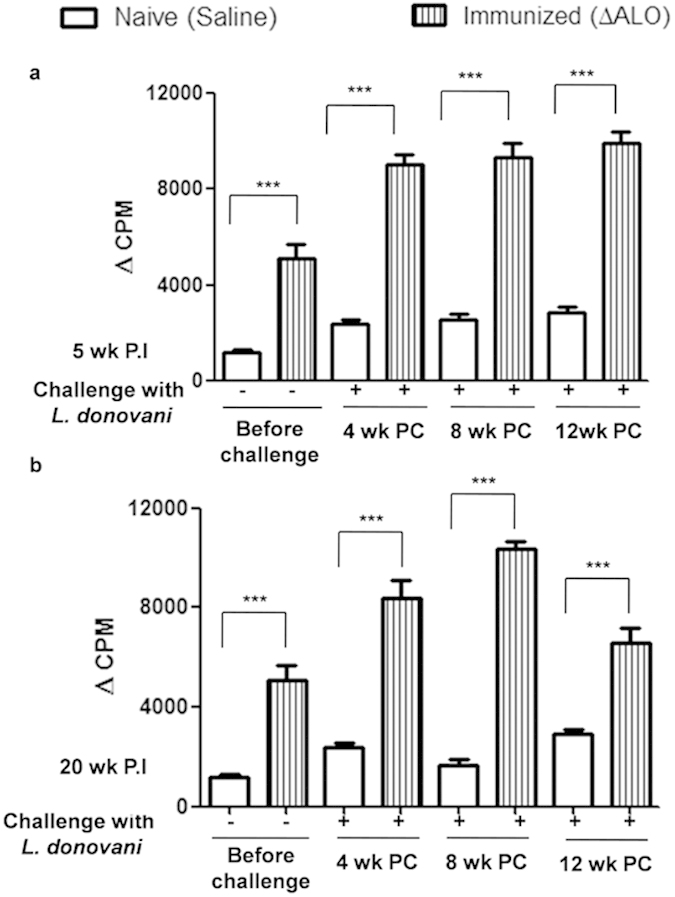
Splenocyte proliferation in saline and *ΔALO* - immunized mice in response to *Leishmania* SLA, pre and postchallenge. Proliferation response to SLA (50 μg/ml) was measured by incorporation of [^3^H]thymidine in both 5 wk (**a**) and 20 wk (**b**) P.I groups. Delta CPM (ΔCPM) represents the difference in counts measured in the presence of antigen compared with that in the corresponding non-stimulated cells in the absence of antigen. Mean and SEM of five mice in each group are shown. The experiment was repeated three times with similar results. *p < 0.05; **p < 0.01; ***p < 0.001 by one way ANOVA with Bonferroni’s multiple comparison post-test. P.C. postchallenge. P.I. postimmunization.

To check the status of T-cell proliferation in the present set of experiment, mice were immunized with saline or *ΔALO*. 5 wk and 20 wk following immunization splenocyte proliferation were analyzed by thymidine incorporation. A ~4 fold high splenocyte proliferation was observed in both the immunized group (5 wk and 20 wk) over the control group in response to SLA (Fig. 4a,b). Immunization was then followed by challenge with WT *L. donovani.* Splenocytes from 5 wk *ΔALO* immunized and challenged mice retained a ~4 fold high proliferative capacity as compared to non-immunized naive challenged mice at all time periods P.C (Fig. 4a).

On the contrary 20 wk P.I mice were able to maintain significantly high proliferative capacity of splenocytes for only initial periods of challenge (4 wk and 8 wk) (Fig. 4b). However at 12 wk P.C the recall response observed in 20 wk P.I (Fig. 4b) mice was less robust as compared to 5 wk immunized mice (Fig. 4a). It is possible that there are no persisting attenuated parasites at 20 wk P.I and the recall response has to be generated from a small number of memory T-cells as compared to a significant number of T-effector cells present during initial period (5 wk) of immunization. In 20 wk immunized animals the increased splenocyte proliferation during initial periods (4 and 8 wk) of challenge may also be attributed to the intrinsic capacity of memory T-cell to mount a rapid secondary immune response during early periods of infection^32^. Overall, these studies suggest that a memory response is maintained over a long period after vaccination with *ΔALO* parasites. Further studies involving adoptive transfer of antigen specific memory T cells from immunized mice to naive mice would be useful to measure the long term immune response generated by *ΔALO* immunization.

**Figure 5:**
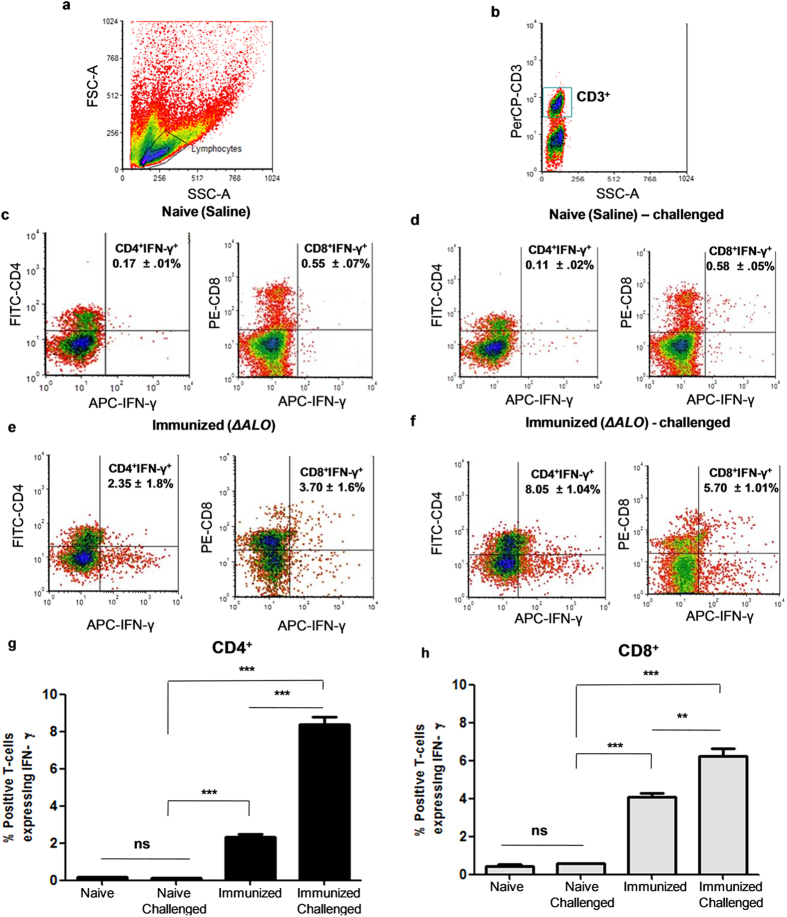
Flow cytometric analysis of *Leishmania* antigen specific IFN- γ - producing CD4^+^ and CD8^+^ T cells in Naive (saline) and *ΔALO* immunized mice, before and 12 wk after challenge. **a-b** represent the common gating steps used in this study are as explained in *Material and Methods* section. Splenocytes from Naive (saline) (**c**), Immunized (*ΔALO*) (**e**), Naive (saline) challenged (**d**) and Immunized (*ΔALO)* challenged (**f**) mice were stimulated with SLA for 48 h and stained for IFN- γ - producing CD4^+^ and CD8^+^ T cells. Values in the quadrants represent the percentage of positive cells. Histograms depicting percentages of IFN- γ^+^ CD4^+^ (**g**) and CD8^+^ (**h**) T cells in various groups. The results are representative of two individual experiments (*n* *=*  5/group). Data represent the mean of triplicate wells ± SD. SSC, Side scatter; FSC, Forward scatter. *p < 0.05; **p < 0.01; ***p *<* 0.001 by one way ANOVA with Bonferroni’s multiple comparison post-test.

Production of IFN- γ in *Leishmania* infection is associated with host capability to prevent the progression of disease. IFN-γ has a pivotal role in the activation of macrophages to kill pathogens and protect the host cell from infection^33^. To evaluate the T cell immune responses, spleens were isolated from BALB/c mice immunized with saline or *ΔALO* and IFN-γ producing CD4^+^ and CD8^+^ T cells were analyzed by intracellular cytokine staining. Splenocytes stimulated with or without SLA were gated first on the basis of forward and side scatter (Fig. 5a) and then on the basis of CD3 expression (Fig. 5b). These cells were then analyzed to check the frequency of cells producing IFN-γ in naive and *ΔALO* immunized mice before (Fig. 5c,e) and after 12 wk of (Fig. 5d,f) challenge. A significant increase in IFN-γ^+^ CD4^+^ (2.35 ± 1.8%) and CD8^+^ T cells (3.70 ± 1.6%) was observed in *ΔALO* immunized mice (Fig. 5e) over IFN-γ^+^ CD4^+^ (0.17 ± 01%) and CD8^+^ (0.55 ± .07%) T cells of naive mice (Fig. 5c). This indicated that *ΔALO* immunization polarizes the T-cells towards IFN-γ production. We further analyzed the IFN-γ expressing CD4^+^ and CD8^+^ T cells in the splenocytes of *ΔALO* immunized and 12 wk P.C mice. Higher percentage of IFN-γ^+^ CD4^+^ (8.05 ± 1.04%) and CD8^+^ (5.70 ± 1.01%) T-cells were observed in *ΔALO* immunized and challenged mice (Fig. 5f) as compared to IFN-γ^+^ CD4^+^ (0.11 ± 0.02%) and CD8^+^ (0.58 ± 0.05%) T-cells of non-immunized naive mice (Fig. 5d) challenged for the same period. Thus, vaccination with *ΔALO* polarizes cellular immune responses towards IFN-γ producing CD4^+^ and CD8^+^ T-cells which further increased after virulent challenge (Fig. 5g,h). In sum, these results indicate that *ΔALO* immunization not only stimulates *Leishmania* antigen specific T- cell proliferation but also induces a strong antigen experienced T cell mediated immune response. Future studies exploring the role of multifunctional CD4^+^ and CD8^+^ T cells in both short- and long-term immunized mice would further characterize the correlates of immune protection induced by *ΔALO* vaccination.

### Resistance to Leishmania infection induced by ΔALO immunization is associated with induction of NO and ROS in splenocytes

**Figure 6:**
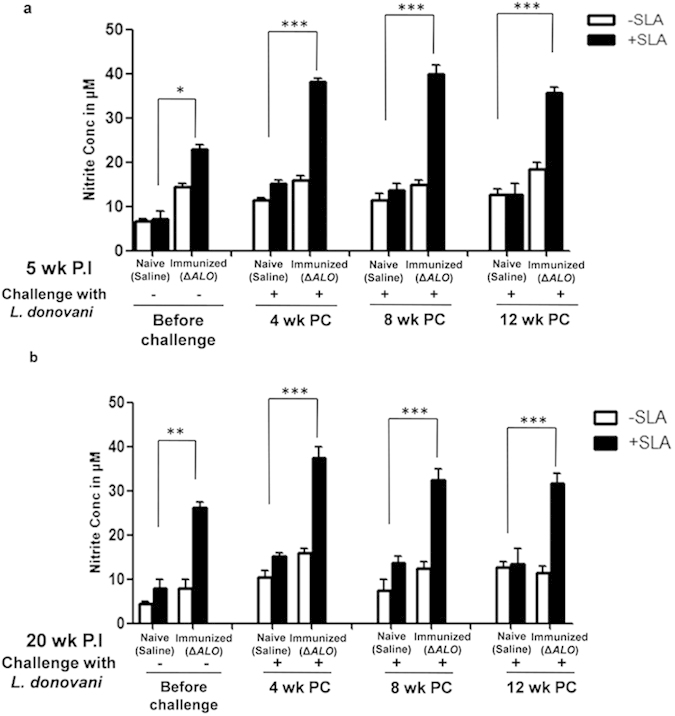
*Leishmania* antigen specific stimulation of NO in the splenocytes of naive (saline), and *ΔALO* immunized mice pre and post various periods of challenge. The activity of NO synthase (NOS_2_), indicated by the amount of released nitrite (NO) in the splenocyte supernatants in presence and absence of SLA was measured by the Griess reaction in both 5 wk (**a**) and 20 wk (**b**) P.I groups. Mean and SEM of five mice in each group are shown. *p < 0.05; **p < 0.01; ***p < 0.001 by one way ANOVA with Bonferroni’s multiple comp**a**rison post-test. P.C. postchallenge. P.I. postimmunization.

**Figure 7:**
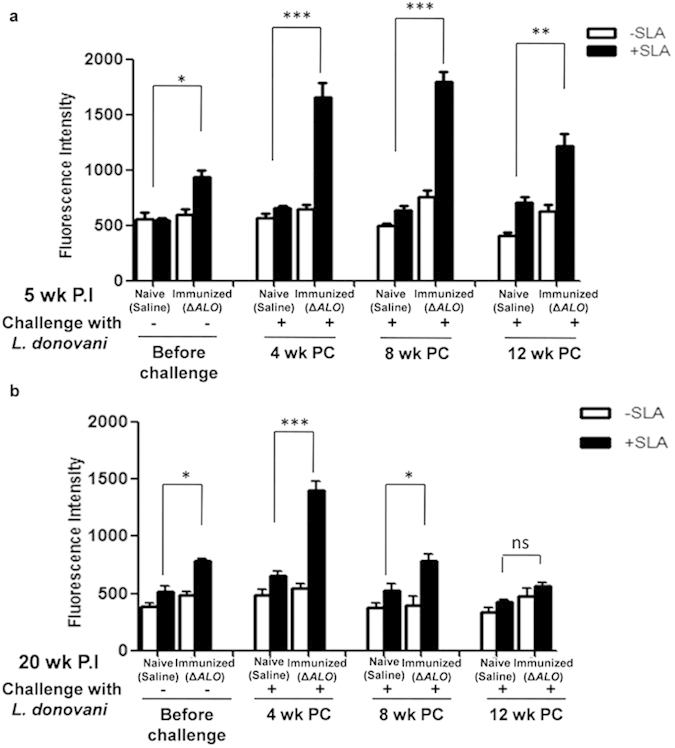
*Leishmania* antigen specific stimulation of ROS in the splenocytes of naive (saline), and *ΔALO* immunized mice pre and post various periods of challenge. ROS generation was measured by H_2_DCFDA probe staining of the splenocytes from 5 wk (**a**) and 20 wk P.I (b) mice in the absence and presence of 50 μg/ml of SLA. Mean and SEM of five mice in each group are shown. *p < 0.05; **p < 0.01; ***p < 0.001 by one way ANOVA with Bonferroni’s multiple comparison post-test. P.C. postchallenge. P.I. postimmunization.

NO and ROS produced by the macrophages are the principal effector molecules required for the intracellular killing of *Leishmania* amastigotes^34^,^35^,^36^. As nitrite and ROS are key factors involved in elimination of *Leishmania*, their levels in different groups were determined. Splenocytes from saline or *ΔALO* -immunized mice were tested for NO and ROS levels (Figs. 6,7) before and after challenge with *L. donovani*.

A substantial increase in *Leishmania* antigen–specific NO (Fig. 6) production was observed in both 5 wk (Fig. 6a) and 20 wk P.I mice (Fig. 6b) as compared to levels in naive mice. However, in both the immunization groups (5 wk and 20 wk) NO production further increased by ~2 fold in *ΔALO* immunized and challenged mice when compared to levels in non-immunized naive challenged mice (Fig. 6a,b). The enhanced nitrite production persisted for up to 12 wk P.C thus indicating long term protection. Thus, the pattern of nitrite induction was found to be similar in challenged animals immunized for both 5 wk and 20 wk.

ROS levels quantitated in splenocytes of both 5 wk and 20 wk P.I mice were considerably higher than that observed for naive mice. ROS production was also analyzed in splenocytes of 5 wk and 20 wk P.I mice at various periods P.C. A ~2 fold higher amount of ROS was maintained at all time points (4 wk, 8 wk and 12 wk), in 5 wk P.I and challenged group as compared to non-immunized naive challenged mice (Fig. 7a). ROS levels in 20 wk *ΔALO* immunized and challenged mice showed ~2 fold higher levels at 4 wk P.C and decreased progressively at both 8 and 12 wk P.C (Fig. 7b).

### Induction of humoral response in ΔALO immunized mice

**Figure 8:**
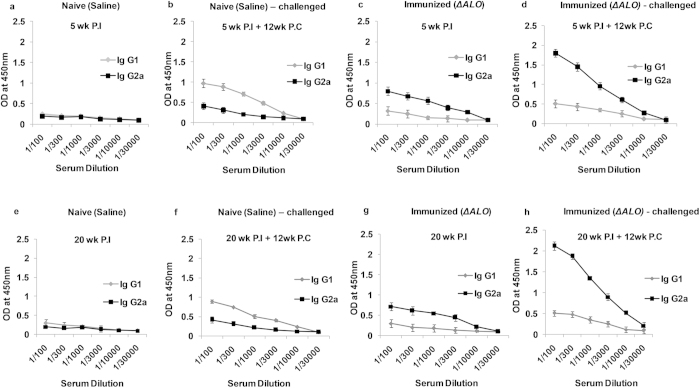
Humoral response induced by *ΔALO* vaccination. ELISA measurement of IgG1, and IgG2a Abs in sera from Naive (saline) (**a,e**), Immunized (*ΔALO*) (**c,g**), Naive (saline) – challenged (**b,f**) and Immunized (*ΔALO*) – challenged (**d,h**) mice. The mice were immunized with *ΔALO* or saline, followed by sera collection at 5 and 20 wk P.I and then subsequently 12 wk P.C.

In the murine VL model, IgG1 and IgG2a class switching is governed by the presence of IL-4 and IFN-γ, respectively^37^. To evaluate humoral responses induced by *ΔALO* immunization, anti-IgG1 and IgG2a antibody titres were measured in mouse sera of naive and *ΔALO* immunized mice, at both 5wk and 20 wk P.I. A substantial increase in IgG2a titres was observed in sera of *ΔALO* immunized mice (Fig. 8c,g) over naive mice (Fig. 8a,e). Twelve weeks after infection, IgG2a titre further increased in the *ΔALO* immunized and challenged mice (Fig. 8d,h) as compared to non-immunized naive challenged mice (Fig. 8b,f). It is well established that titres of IgG1 antibody increase with *L. donovani* parasite load^38^. A decrease in IgG1 titre in the immunized challenged group (Fig. 8d,h) as compared to the non-immunized naive challenged mice (Fig. 8b,f), correlates with the decreasing parasite loads after immunization. Thus, immunization with *ΔALO* led to a selective increase in Th1-driven IgG2a antibody levels accompanied by decrease in IgG1 titres. *ΔALO* immunization results in similar humoral responses at both 5 wk and 20 wk P.I.

## Discussion

The development of a safe, effective and affordable antileishmanial vaccine is a critical global public-health priority. No vaccine is currently available and those tested so far have been disappointing in field studies. Thus, it is imperative to develop alternative priming strategies which could induce immunological memory against *Leishmania*. Based on prior knowledge that individuals who recover from natural *Leishmania* infection are protected from re-infection and develop life-long protection, genetically altered live attenuated parasites with controlled infectivity could be explored as potential vaccine candidates. In addition, live attenuated parasites provide a complete plethora of parasite antigens to generate a diverse antigen-specific memory immune response that is important for protection against infection.

In the present study, we evaluated the protective efficacy of ascorbic acid-deficient live mutants of *Leishmania donovani* against VL. Ascorbate is known to be an important anti-oxidant in most eukaryotes, which can directly metabolize ROS and mediate electron transfer to ascorbate-dependent peroxidases^39^. Ascorbate peroxidase has been reported earlier to have a role in anti-oxidant defense system in *Leishmania*^40^,^41^. Further, it has been found to play a role in controlling parasite differentiation and survival in host macrophages^42^. Arabino-1, 4-lactone oxidase (ALO) enzyme from *L. donovani* catalyzes the last step in ascorbate biosynthesis pathway. We have earlier demonstrated that *ΔALO* attenuation debilitates the parasite by making it susceptible to oxidative defence mechanisms of the host cells^18^. Here, we show that live attenuated *ΔALO* parasites are safe, induce protective immunity and can provide sustained protection against *Leishmania donovani.* We further report that host protection correlates with the induction of a *Leishmania* antigen specific cell mediated immune response.

The virulence of *ΔALO* parasites was first checked in mice. These attenuated parasites are unable to survive beyond 16 weeks in susceptible BALB/c mice thus making it safe for its use as a vaccine candidate. We did not observe any parasites in the liver and spleen after dexamethasone treatment in *∆ALO*-infected mice at 20 wk post-infection. These observations in dexamethasone treated immunosuppressed mice further reinforce its safety even in the immune compromised host. Earlier reports using p27 gene knockout parasites also showed that the parasites can persist for an extended period without causing pathogenesis^15^.

*ΔALO* mutants were further dissected for their protective efficacy against VL in BALB/c mice model. The efficacy of the *ΔALO* parasites as immunogen was confirmed by significant parasite control in immunized mice post-infection at both early (5 wk) and late (20 wk) stages of immunization. *ΔALO* immunization resulted in sterile protection in liver and significantly reduced parasite burden in spleen as compared to the naive challenged mice. The dichotomy of immune response between liver and spleen has been reported earlier^43^.

It is generally accepted that for any vaccine to be successful against VL, it should skew the immune response toward the Th1 type^44^. IFN-γ is the principal Th1 effector cytokine which is known to direct the immune response toward a Th1 phenotype^45^.Therefore the secretion of IFN-γ was analyzed in splenocytes of vaccinated and control group of mice, before and after various periods of challenge. Besides IFN-γ we also checked the induction of other decisive cytokines like IL-12 and TNF-α. IL-12 is required for initiation as well as maintenance of a Th1 cellular response thus providing resistance to leishmaniasis^46^. In addition, along with IFN-γ, TNF-α has also been reported to induce leishmanicidal activity in macrophages^47^,^48^. Thus, enhanced levels of these Th1 cytokines have a synergistic role in killing the parasite. Our results collectively show that *ΔALO* immunization conferred protective immunity in both short term (5 wk) and long term (20 wk) immunized mice, by triggering production of Th1cytokines like IFN-γ, TNF-α, and IL-12 in the immunized-challenged group as compared to naive controls infected for similar time period. Overall the level of Th1 cytokines were lower in 20 wk immunized and challenged mice as compared to that observed for the 5 wk immunized and challenged group. It is possible that in the 20 weeks immunized mice, since there are no persisting live attenuated parasites, the immune response is generated from a small number of memory T cells as compared to significant number of T-effector cells present during early stages (5 wk) of immunization.

We have reported earlier that infection with *ΔALO* parasites modulates the redox homeostasis by increasing the ROS and NO levels of host cells^18^. In the present study, 20 wk immunized and challenged animals showed decline in ROS levels over a period of time. However, NO levels remained high at all time periods P.C (Fig. 6b). These findings are consistent with previous reports which suggest that ROS is one of the initial host defence response produced due to innate stimuli^49^. Thus, it may be possible that after 20 wk of immunization when these attenuated parasites are cleared from spleen of mice ROS levels decline due to decrease in innate stimulus. In contrast to ROS, NO is produced in response to immune stimuli particularly cytokines^49^. Thus, overall the sustained secretion of NO by splenocytes of 20 wk *ΔALO* immunized and challenged mice indicates a pro-inflammatory cytokine dominating milieu in *ΔALO* immunized mouse spleens, which in turn favours the host in clearing parasites. This result further corroborates our previous observation that *ΔALO* immunization primes a selective long-term Th1 governed pro-inflammatory immune response.

Correlates of protection was further indicated by increased percentage of IFN-γ producing CD4^+^ and CD8^+^ T cells in immunized challenged mice as against naive controls. It is well established that both CD4^+^ and CD8^+^ T cells are the primary source of IFN-γ^50^. CD8^+^ T cells contribute to the control of pathogens by cytokine production, cytolytic activity or both. The cytolytic activity of these cytotoxic T-lymphocytes (CTL) was not analyzed and needs to be evaluated. Further studies are also required to analyze and characterize the role of memory T-cells in *ΔALO* immunization.

In the present study, a robust IgG2a antibody response was also observed in both the immunized groups (5wk and 20 wk P.I) after challenge. These results are consistent with enhanced production of IFN-γ in vaccinated animals because IFN-γ directly regulates IgG2a class switching^45^. Higher level of IgG2a might also contribute to pathogen clearance in vaccinated animals^51^. Thus, our data suggests that *ΔALO* vaccination stimulates all arms of protective immune response to achieve significant protection against VL.

In summary, this study characterizes, live attenuated *ΔALO* mutants as candidate vaccine that has been evaluated for its *in vivo* persistence, immunological response, and protective efficacy. Overall, our results show that *ΔALO* mutants are highly immunogenic and confer a significant degree of protection in BALB/c mice against virulent *L. donovani* challenge. *ΔALO* parasites elicit a strong antigen specific cell mediated immune response in both the presence and the absence of parasite. Our report for the first time shows that intracellular parasites attenuated in their anti-oxidative defence mechanism can be exploited as vaccine candidates. Future studies using higher animal like hamsters and nonhuman primates and also evaluating a more favourable route of administration need to be tested.

## Methods

### Animals

6-8 wk old female BALB/c mice from the National Institute of Nutrition (Hyderabad, India) were used in the experiments. The study was performed in strict accordance with the guidelines by the Committee for the Purpose of Control and Supervision of Experiments on Animals (CPCSEA), Ministry of Environment and Forest, Government of India. The protocol was approved by the Institutional Animal Ethics Committee (IAEC) of Jawaharlal Nehru University (JNU) (IAEC Code Number: 11/2013).

### Parasite Culture

*L. donovani Bob* strain (*LdBob* strain/MHOM/SD/62/1SCL2D) (wild type, WT) was maintained in female BALB/c mice. Amastigotes isolated from infected spleen were then transformed to promastigotes in Medium 199 (Sigma-Aldrich) supplemented with 100 μg/ml streptomycin, 100 U/ml penicillin (Sigma-Aldrich), and 30% FBS (FBS, Hyclone, U.K). Freshly transformed promastigotes were used for infecting BALB/c mice. For experiments with virulent promastigotes, parasites were maintained in liquid culture for no longer than 3 wk after isolation from Balb/C mice. *ALO* null mutant cell line of *L. donovani* (*ΔALO*) were cultured at 22 °C in M199 medium (Sigma) supplemented with 0.13 mg ml^-1^ each of penicillin and streptomycin (Sigma) and 5% heat-inactivated fetal bovine serum (FBS, Hyclone, U.K). The parasites were in addition maintained in 150 μg ml^−1^ hygromycin and 300 μg ml^−1^ paromomycin.

### Immunizations and challenge studies

For the vaccination and protection studies mice were randomly divided into two groups and given treatment for 5 wk (n = 60) and 20 wk (n = 60). Four different treatments were administered to mice with in each group. These sub-groups were: a. Naive mice (n = 5), b. Naive challenged mice (n = 25), c. *ΔALO* immunized (n = 5), d. *ΔALO* immunized and challenged mice (n = 25). Five mice from each sub-group were utilized for the analysis of immune response at different time periods (i.e. before challenge, 4 wk, 8 wk and 12 wk P.C). Additionally, the parasite load was monitored in the above mentioned groups and also at 16 and 20 wk postchallenge. Immunization of mice was done via tail vein with 3 × 10^6^ stationary-phase *ΔALO* parasites and challenged with 1 × 10^7^ virulent *L. donovani* promastigotes.

For the immune-suppression study, mice were infected with either WT or *ΔALO* parasites, and at 20 wk post-infection, 2 mg/kg dexamethasone sodium phosphate (Sigma-Aldrich) in PBS was administered s.c. three times per wk^52^. At the end of this treatment, mice were sacrificed and visceral organs evaluated for parasite burden.

### Estimation of parasite load

After various periods P.C, parasite load was measured in spleen and liver from the *L. donovani*–challenged mice by limiting dilution as previously described^19^. Briefly, the organs were homogenized, resuspended in RPMI medium and submitted to serial dilutions in 96 well plates. The number of parasites per gram of organ was calculated as previously described^19^. As an additional confirmation for the presence of parasites in tissues, real-time PCR was done as reported earlier^53^. 100 ng of total DNA was isolated from spleen and liver of each mouse and was used as template for the real-time PCR.

### Source of soluble antigen (SLA)

SLA was prepared from stationary phase *L. donovani* (*Bob*) promastigotes. Briefly, cells were harvested by centrifugation at 4000 *g* for 15 min at 4 °C followed by washing with PBS and were then resuspended in PBS containing protease inhibitors. Cell lysis was done by sonication with six pulses of 30 s each (MSE Sonicator). The lysate was centrifuged at 17,000 *g* for 15 min at 4 °C.

### Cytokine assays

The concentrations of IFN-γ TNF-α, IL-12, IL-4, and IL-10 in culture supernatants were determined by ELISA. Briefly, spleen cells from BALB/c were isolated from each group of mice at different intervals and resuspended in RPMI 1640 medium supplemented with 10% FBS. Splenocytes were pooled and incubated in a 96-well flat-bottom plate (Nunc, Roskilde, Denmark) at a density of 5 × 10^5^ cells/well and cultured with SLA (50 μg/ml). After 48 h, supernatants were collected and diluted serially and cytokine concentrations were quantitated using ELISA. The assay was performed using a BD Pharmingen Opt EIA kit according to the manufacturer’s instructions.

### Cell proliferation assay

Spleens from mice of each group were removed aseptically on a sterile dish containing RPMI 1640 medium (Sigma). Single-cell suspensions were prepared by grinding the spleen with the disk bottom of the plunger of a 10-ml syringe. RPMI 1640 medium (5–10 ml) was added to the suspension and the contents were mixed well. The dish was kept undisturbed for 2 min and the clear supernatant was pipetted out slowly. Cells were pelleted by centrifugation at 4 °C at 250 *g* (Sorvall RC-5 centrifuge, HB-4 rotor) for 10 min. The pellet was washed once with 0.9% ice-cold ammonium chloride to lyse the erythrocytes. The remaining cells were resuspended to a density of 2.5 × 10^6^ cells/ml in RPMI 1640 containing 10% FBS and then divided into 200-μl aliquots (5 × 10^5^ cells) in flat-bottom 96-well plates (Nunc). Cells were incubated in the presence or absence of SLA (50 μg/ml) and incubated for 3 days at 37 °C in a 5% CO_2_ incubator. Proliferation was measured by incorporation of 1 μCi of [^3^H]thymidine (Amersham Biosciences) over the last 16 h of the culture. Incorporation of [^3^H]thymidine was measured by a beta scintillation counter (PerkinElmer 1450 LSC and Luminescence Plate Counter). All assays were performed in triplicate.

### Intracellular cytokine staining

The assay was performed using Cytofix/Cytoperm Plus Fixation/ Permeabilization kit (BD Biosciences). Briefly, splenocytes from mice with saline control (Naive group), immunized with *ΔALO* (Immunzation group), Naive- challenged group and Immunized-challenged group were recovered, resuspended in RPMI 1640 medium supplemented with 10% FBS, and incubated in a 96-well flat-bottom plate at a density of 2 × 10^6^ cells/well. The cells were stimulated with 50 μg/ml SLA or PMA/ionomycin (as a positive control) or without antigen (as a negative control). After 4 h of stimulation/ incubation at 37 °C, brefeldin A (1 μg/ml, GolgiPlug) was added to each well and the incubation was resumed for an additional 12 h at 37 °C. Cells were blocked with 1 μg/ml BD Biosciences Fc Block for 15 min at 4 °C and then stained with PerCP-anti-mouse-CD3e (BD Biosciences), FITC-anti-mouse-CD4 (BD Biosciences), and PE-antimouse- CD8 (BD Biosciences) or their respective isotype controls (BD Biosciences) in 50 μl of staining buffer for 30 min at 4 °C. Cells were permeabilized and fixed using a BD Biosciences Cytofix/Cytoperm kit. Intracellular staining was then performed according to the protocol provided by BD Biosciences. Allophycocyanin-anti-mouse-IFN-γ (BD Biosciences) or its respective isotype control was used for intracellular staining of the cells. Finally, cells were analyzed on a FACS, BD Biosciences FACS Calibur system using BD Biosciences CellQuest software. Spleen cells were gated based on forward and side scatter to select only lymphocyte population devoid of dead cells. CD3^+^ T-cells from this lymphocyte population were further gated for CD4^+^ and CD8^+^ T-cells which were then analyzed for IFN- γ expression.

### Quantification of NO

Splenocytes (5 × 10^5^ cells/well) from different groups of experimental mice were incubated in a 96-well plate with or without SLA (50 μg/ml) in a CO_2_ incubator at 37 °C. After 48 h the culture supernatant was collected and analyzed for their nitrite content using Griess reagent as described earlier^18^. Concentration of nitrite (NO2^-^) was determined using the standard curve prepared from the known concentration of sodium nitrite.

### Measurement of reactive oxygen species (ROS)

The cell-permeable fluorescent probe H_2_DCFDA was used to measure the levels of ROS. Splenocytes (5 × 10^5^) from different groups of mice were incubated in a 96-well plate with or without SLA (50 μg/ml) at 37 °C in a CO_2_ incubator. After 72 h H_2_DCFDA (1 μg/200 μl/well) was added at room temperature for 20 min. Relative fluorescence was measured by using an excitation wavelength of 504 nm and emission wavelength of 529 nm in a Microplate Reader. Fluorometric measurements were made in triplicate and expressed as fluorescence intensity units.

### Measurement of anti- IgG

Pooled mouse serum from each group of mice was used to assay the antibody titres. Serum Ig isotypes were assayed by ELISA using 2 μg/ml of SLA. Briefly, 96- well plates were coated with SLA in 100 μl of carbonate buffer (pH 9.2) and incubated overnight at 4 °C. Plates were blocked with 1.5% BSA in PBST (0.05% Tween 20) at 37 °C to prevent nonspecific binding. Mouse serum (100 μl) diluted to a concentration of 1/100 –1/30,000 in PBST was then added to the wells. Plates were further incubated with biotinylated-conjugated rabbit anti-mouse IgG1 and IgG2a and streptavidin-conjugated HRP. Color was developed by incubation with *o*-phenylenediamine and 30% H_2_O_2_. The absorbance was determined at 490 nm.

### Statistical analysis

The statistical analysis was made using the GraphPad Prism software (version 5.0 for Windows). Statistical analysis with the vaccinated and/or infected mice was performed by one-way analysis of variance (ANOVA), using the Bonferroni’s post-test for multiple comparisons of groups. Differences were considered statistically significant when *p* < 0.05. Data is represented as mean ± SD. The results are representative of three independent experiments with similar results.

## Additional Information

**How to cite this article**: Anand, S. and Madhubala, R. Genetically Engineered Ascorbic acid-deficient Live Mutants of Leishmania donovani induce long lasting Protective Immunity against Visceral Leishmaniasis. *Sci. Rep.* **5**, 10706; doi: 10.1038/srep10706 (2015).

## Supplementary Material

Supplementary Information
