# Supplementary material for: Genetically Engineered Ascorbic acid-deficient Live Mutants of Leishmania donovani induce long lasting Protective Immunity against Visceral Leishmaniasis
Source: Sci Rep. 2015 Jun 2;5:10706. doi: 10.1038/srep10706 (PMC4451804; doi:10.1038/srep10706)
Supplement: Supplementary Information [file srep10706-s1.pdf]

SUPPLEMENTARY INFORMATION

Genetically Engineered Ascorbic acid-deficient Live Mutants of Leishmania donovani induce long lasting Protective Immunity against Visceral Leishmaniasis

Sneha Anand and Rentala Madhubala*

*School of Life Sciences, Jawaharlal Nehru University, New Delhi 110067, India

Supplementary Table S1: Real time PCR confirmation of parasite presence in spleens and livers of mice infected with different parasites. The data presented are representative of two experiments with similar results.

S.No.	Groups	Ct Values[a,b]	
		**Liver**	**Spleen**
1.	Naive Mice	37.3 ± 0.8	36.2 ± 0.7
2.	5 wk post-infection with WT	15.4 ± 1.2	16.5 ± 1.1
3.	5 wk post-infection with *ΔALO*	27.1 ± 1.13	24.6 ± 1.2
4.	16 wk post-infection with WT	20 ± 1.12	17.1 ± 1.21
5.	16 wk post-infection with *ΔALO*	36.1 ± 1.16	34.1 ± 1.7

S.No.	Controls	Ct Values
1.	Reactions without g DNA	37.2 ± 0.63
2.	Positive control, 250 pg, DNA from *L. donovani* parasites	11.38 ± 0.7

[a] The experiment was repeated separately for each mouse in a group and data is shown as Mean ± SEM.
[b] 100 ng of total DNA from spleen and liver of each mouse was used as template

Supplementary Table S2: Real time PCR confirmation of parasite presence in spleens and livers of mice immunized and challenged for various time periods. The data presented are representative of two experiments with similar results.

S.No.	Groups	Ct Values[a,b]	
		**Liver**	**Spleen**
1.	5 wk naive-challenged (12 wks P.C)	19 ± 1.3	20.18 ± 1.1
2.	5 wk Immunized-challenged (12 wk P.C)	34.6 ± 1.6	26.4 ± 1.81
3.	5 wk Immunized-challenged (16 wk P.C)	35.2 ± 1.44	29.3 ± 1.56
4	5 wk Immunized-challenged (20 wk P.C)	36.0 ± 1.44	29.8 ± 1.9
5.	20 wk naive-challenged (12 wk P.C)	18.1 ± 2.43	16.9 ± 1.56
6.	20 wk Immunized-challenged (12 wk P.C)	36.2 ± 1.8	22.3 ± 1.56
7.	20 wk Immunized-challenged (16 wk P.C)	36.1 ± 1.44	26.3 ± 1.56
8.	20 wk Immunized-challenged (20 wk P.C)	37.1 ± 1.21	28.8 ± 2.06

S.No.	Controls	Ct Values
1.	Reactions without g DNA	37.2 ± 0.63
2.	Positive control, 250 pg, DNA from *L. donovani* parasites	11.38 ± 0.7

[a] The experiment was repeated separately for each mouse in a group and data is shown as Mean ± SEM.
[b] 100 ng of total DNA from spleen and liver of each mouse was used as template
